# Immunogenicity and Safety of Influenza Vaccination in Systemic Lupus Erythematosus Patients Compared with Healthy Controls: A Meta-Analysis

**DOI:** 10.1371/journal.pone.0147856

**Published:** 2016-02-04

**Authors:** Zhengfa Liao, Hao Tang, Xiaojia Xu, Yaping Liang, Yongzhen Xiong, Jindong Ni

**Affiliations:** 1 Department of Epidemiology and Biostatistics, Guangdong Medical University, Dongguan, China; 2 School Clinic, Guangdong Medical University, Dongguan, China; Renal Division, Peking University First Hospital, CHINA

## Abstract

**Objective:**

To assess the immunogenicity and safety of influenza vaccine in patients with systemic lupus erythematosus (SLE).

**Methods:**

Relevant articles were retrieved from electronic databases. Seroprotection rate, seroconversion rate and factors that increase antibody geometric mean titer (GMT) were used as indices to measure the immunogenicity. The safety of vaccine was assessed through monitoring adverse events, which included side effects and SLE exacerbations. We performed a meta-analysis of influenza vaccine seroprotection, seroconversion and adverse effects. SLE exacerbation after vaccination was comprehensively described. We used the Committee for Proprietary Medicinal Products (CPMP) guidelines to determine whether influenza can induce adequate immunogenicity in patients with SLE.

**Results:**

Eighteen studies with 1966 subjects met the inclusion criteria. At least 565 of the subjects were patients with low-to-moderate SLE Disease Activity Index (SLEDAI) score or stable SLE disease. Compared with the general population, seroprotection rate in SLE patients was significantly decreased in patients with H1N1 [odds ratio (OR) = 0.36, 95% confidence interval (CI): 0.27–0.50] and H3N2 vaccination (OR = 0.48, 95% CI: 0.24–0.93), but not influenza B vaccination (OR = 0.55, 95% CI: 0.24–1.25). Seroconversion rate also significantly decreased in patients with H1N1 (OR = 0.39, 95% CI: 0.27–0.57) and influenza B (OR = 0.47, 95% CI: 0.29–0.76) vaccination, but not H3N2 vaccination (OR = 0.62, 95% CI: 0.21–1.79). However, the immunogenicity of influenza vaccine in SLE patients almost reached that of the CPMP guidelines. The OR for side effects (patients versus healthy controls) was 3.24 (95% CI: 0.62–16.76). Among 1966 patients with SLE, 32 experienced mild exacerbation of SLE and five had serious side effects for other reasons.

**Conclusion:**

Influenza vaccine has moderate effect on protecting patients with SLE. The side effects of influenza vaccine are not serious and are manageable. With consideration of a higher risk of SLE exacerbation and a more severe course of infection among SLE patients, influenza vaccination should be promoted among SLE patients with a low-to-moderate SLEDAI score or stable disease.

## Introduction

Systemic lupus erythematosus (SLE) is an autoimmune disease characterized by dysregulation of the immune system and secretion of autoantibodies. The autoantibodies combine with autologous components to form complexes that are deposited in tissues and impair various organs. SLE injures several organs and impairs immunity. The reciprocal relationship between SLE and infection is of importance. Patients with SLE are susceptible to infection because of impaired immunity. Infections are major causes of morbidity and mortality in patients with SLE, accounting for 20%–50% of deaths [1]. SLE symptoms can be elicited and exacerbated by infection [2, 3].

Influenza leads to 1.14–6.24 million hospital admissions and 4910–27,170 deaths annually in the US [4]. The influenza vaccination has been proved the most effective, economic and safe method to prevent influenza among healthy populations. It is doubtful that this is the case among patients with SLE because of their immunosuppressive therapy and the disease itself.

Only a few isolated studies have investigated immunogenicity of influenza vaccine in SLE patients. However, the results of these studies were not consistent. Some studies reported that no difference was found in the immunogenicity between patients with SLE and healthy controls, whereas other studies demonstrated lower efficacy of influenza vaccine in SLE patients than in healthy control.

The safety of influenza vaccine in SLE patients is disputed. A few studies have reported SLE flares and deaths emerged after vaccination. Vaccines prevent infectious diseases through stimulating the immune system to produce specific antibodies. Flares experienced by SLE patients also involve stimulation of the immune system, although it is triggered by unknown factors. The flares are considered to one of the following: introduction of a new treatment in the presence of worsening of an already active system or in response to the activation of a new system; an increase in drug dose for the above reason [5]. As a result, it is proposed that the influenza vaccine may be a culprit in aggravation of SLE. These facts are disturbing to patients and enough to turn them away from vaccination.

Many SLE patients have unstable disease. Physicians usually do not advocate influenza vaccination in these patients. The aforementioned doubts may have created a barrier for physicians advising their patients to accept the influenza vaccine. Lack of physicians’ advice and patients’ worries about the safety and efficacy of influenza vaccine have contributed to a low rate of influenza vaccination among SLE patients [6]. To eliminate the doubts about the safety and immunogenicity of influenza vaccine in patients with SLE, we conducted a meta-analysis that investigated the safety and immunogenicity of influenza vaccine. The conclusions of our study can benefit clinical practice and public health strategy.

## Methods

### Retrieval strategy

We searched PubMed, Embase, Cochrane Library, China National Knowledge Infrastructure (CNKI), and Science Direct databases for articles published before April 2015. We searched PudMed and the Cochrane Library using the subject headings ‘lupus erythematosus, systemic’ and ‘influenza vaccine’. In Embase, the search formula was ‘systemic lupus erythematosus’/exp or ‘systemic lupus erythematosus’ and (‘influenza vaccine’/exp or ‘influenza vaccine’ or ‘influenza’/exp or ‘influenza vaccination’). Key words such as ‘influenza vaccine’, ‘influenza vaccination’, ‘influenza immunity’, or ‘influenza immunogenicity’ in combination with ‘systemic lupus erythematosus’, ‘systemic lupus erythematosus’ or ‘SLE’ were used in Science Direct and CNKI. We also manually searched the references of the retrieved articles for further studies.

### Eligibility and exclusion criteria

The eligibility criteria were: studies of SLE patients who received the influenza vaccine; study outcome was the titer of antibody against influenza virus which was measured by hemagglutination-inhibition (HAI) test; and cohort study design. Exclusion criteria were: study sample size < 10; control group were unhealthy people; no clear data in the abstract; and duplicated data. We assessed the quality of the articles that met the eligibility criteria using the Newcastle—Ottawa Scale for cohort studies. The assessment was independently completed by two researchers. When there was a discrepancy in the number of stars in one section, consensus was reached after discussion between the two researchers.

### Data abstraction

Before data abstraction, our group discussed what information could be obtained from the literature. We designed a questionnaire to survey the information that would be needed. All articles were analyzed by at least two researchers and the questionnaires were completed after the analysis. If there were some controversial views, a third investigator arbitrated the conflict. Regarding influenza vaccine immunogenicity, factors that increase antibody GMT, seroprotection and seroconversion rates were extracted from the study. Seroprotection was defined as a serum HAI titer of not less than 1:40 after vaccination, and seroconversion was defined as a titer below 1:10 before vaccination rising to at least 40, or a titer over 1:10 increasing >4-fold after vaccination. Seroconversion is used widely as an index for vaccine efficacy. Titers no less than 1:40 can be viewed as protective in healthy adults [7]. Seroconversion and seroprotection rates are the percentages of recipients who meet the definition of seroprotection and seroconversion. If the original studies did not define seroprotection and seroconversion, the above definition would be applied in those studies.

We also considered information that might have affected the influenza antibody titer. The information included age of subjects, interval between vaccination and serology, drug treatment, and the virus vaccine strain. We also monitored the adverse effects and abnormal changes in disease activity.

### Data analysis

The HAI test was used to quantitate the influenza antibodies. The CPMP guidelines were adopted to determine whether influenza vaccine induces adequate immunity among patients with SLE [8]. The CPMP guidelines state that cut-off levels of vaccine immunogenicity for the general population are seroprotection rate >70%, seroconversion rate >40%, and factors that increase antibody GMT >2.5-fold. To meet the CPMP guidelines, each of the vaccine antigens must meet at least one of the above criteria [9]. In order to be close to the real-life situation, the crude rates were calculated to compare the guidelines. ORs were used to evaluate the difference in immunogenicity between two groups. The ORs with exact binomial 95% CIs were displayed in forest plots.

Most studies determined the safety of vaccination in SLE patients through monitoring adverse events. The latter included side effects of the vaccine and SLE exacerbation after vaccination. The local side effects included pain, redness, swelling, and itching. The systemic side effects included arthralgia, fever, headache, myalgia, sore throat, cough, diarrhea, rhinorrhea and nasal congestion. Severe side effects were defined as those requiring hospitalization and resulting in death. Because no SLE exacerbation emerged in general population after vaccination, only the rate of side effects in the SLE patients was compared with that in general population. The SLE exacerbations were comprehensively described in this article. The SLE Disease Activity Index (SLEDAI or SLEDAI-2K) was used to evaluate disease status [5, 10], and changes in disease status correlated with disease deterioration. Flares for SLE were also considered to be disease exacerbation.

Before pooling the outcomes, we presumed that heterogeneity was not significant when the *p* value of Cochrane’s *Q* test was not <10% [11]. Selection of the fixed- or random-effects model depended on the result of Cochrane’s *Q* test. When the heterogeneity was significant, the Mantel—Haenszel random model was used. If not, we preferred the Mantel—Haenszel fixed model [12]. Publication bias was assessed through visual inspection of funnel plot asymmetry. Asymmetry was also tested by Egger’s linear regression analysis [13]. Statistical analysis was performed using Revman version 5.2 (provided by the Cochrane Collaboration).

## Results

### Literature review

**Fig 1:**
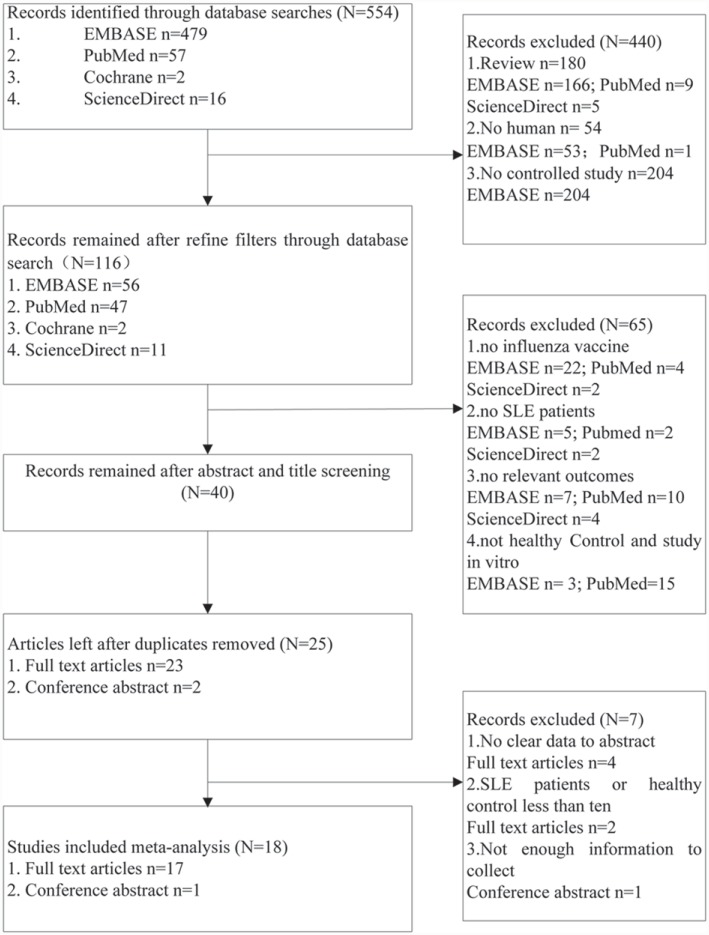
Flow Chart of Study Selection.

Five hundred and fifty-four relevant articles were identified from four databases (Fig 1). The studies were selected in the sequence of title, abstract and full text. After screening, there were 18 eligible studies that included 1966 SLE patients and 1112 healthy individuals. The included studies comprised 17 full-text articles and one conference abstract. Although the conference abstract was not in possession of enough information, it met the need for abstracting data. All the studies were published in 1978–2013, and were cohort studies comparing with SLE patients with healthy population. The quality of all studies was not < 5 stars.

### Description of included studies

Six studies were from Brazil, four from the US, three from the Netherlands, and one each from Mexico, Italy, Israel, Taiwan and Poland (Table 1).

**Table 1:** Description of Characteristics of Included Studies.

Study	Country	M/F	Age (range or M±SD)	Diease activity at vaccination (SLEDAI range or M±SD)	Factor increase in GMT			Adverse event	
					H1N1	H3N2	B	SLE patients	Healthy people
Aikawa, 2012 [14]	Brazil	-	(9,21)	-	12.9	-	-	-	L_21_, S_27_
Brodman, 1978 [15]	AU	2/44	36	No limitation	6.8	-	-	S_9_, D_11_	S_9_
Del, 2006 [16]	Italy	1/13	43.42±12.18	6.21±2.45	13.5	19.9	4.0	D_2_	L_2_
Elkayam, 2011 [17]	Israel	4/17	41.7 ±11.5	0.9±1	10.26	-	-	L_3_, S_19_	L_1_,S_1_
Holvast, 2006 [18]	Netherlands	6/50	(18,78)	(0,5)	4.38	3.66	3.95	L_39_, S_16_	L_9_,S_4_
Holvast, 2009a [19]	Netherlands	10/44	44.8 ± 13.6	(0,8)	4.0	5.5	-	-	-
Holvast, 2009b [20]	Netherlands	9/43	45.2±10	(0,4)	2.7	2.1	1.9	-	-
Louie, 1978 [21]	US	1/11	34.09±10.89	No limitation	-	-	-	D_1_	-
Lu, 2011 [22]	Taiwan	1/20	34.3±11.8	(0,7)	5.26	-	-	L_0_, S_0_, D_1_	L_0_, S_0_
Ristow, 1978 [23]	US	1/28	(19, 67)	No limitation	6.96	-	-	L_7_, S_2_, D_3_	L_0_, S_0_
Saad, 2011 [24]	Brazil	-	-	-	7.89	-	-	-	L_33_, S_60_
Wallin, 2009 [25]	Brazil	1/46	40.57±9.9	1.19±2.0	5.66	6.17	3.65	-	-
Wiesik, 2010 [26]	Poland	3/59	(18, 67)	4.8±5.1	6.23	-	-	L_0_, S_4_, D_7_	L_4_, S_1_
Campos, 2013 [27]	Brazil	27/91	16± 3.5	6.0±5.8	8.1	-	-	L_33_, S_35_	L_2_, S_5_
Borba, 2012 [28]	Brazil	41/514	36.7±12.2	3.2±3.9	8.0	-	-	L_48_, S_142_	L_30_, S_47_
Borba, 2010 [29]	Brazil	-	-	3.5 ± 4.2	-	-	-	D_12_	-
Mercado, 2004 [30]	Mexico	18/0	34.7	5.6 ± 4.5	3.61	3.74	3.47	L_1_, S_1_	L_0_, S_0_
Long, 2012 [31]	US	4/16	12.7±3.4	-	-	-	-	-	

Note: -, no mention or not applicable; F/M, Female/male; S_n_, number of systemic side effects; L_n_, number of local side effects; D_n_, number of SLE exacerbation and serious adverse events.

Complete information about SLE patients’ age was reported in 15 full-text articles. Two of the remaining studies did not have specific information about the age of SLE patients, and the conference abstract did not have any detailed information about the study subjects. In the 17 full-text articles, the study subjects were adult patients with SLE (mean age ≥18 years), except for three studies which recruited patients with juvenile SLE.

The subjects in all studies had drug treatment that may have affected the vaccine response. According to subgroup analysis in the original studies, seven studies showed that drugs such as glucocorticoid, azathioprine, methotrexate and mycophenolate attenuated vaccine immunogenicity. It was concluded in two studies that only hydroxychloroquine can enhance influenza immunity among SLE patients. Three studies demonstrated that drug treatment had no influence on vaccine response. The remaining ten studies did not mention the effect of drugs on vaccine response.

Information on SLE disease activity was gathered from 13 full-text articles. Three of those studies selected their subjects without limitation on SLE disease status. The SLEDAI scores of patients before vaccination were recorded in the remaining 10 studies. A total of 1106 patients with low-to-moderate SLEDAI score or stable disease were reported in the original studies. However, disease activity of the remaining patients was not clear.

Trivalent, bivalent and univalent influenza vaccines were used in the studies (data not show). Seven studies utilized trivalent influenza vaccine that contained the surface antigens of the H1N1, H3N2 and B influenza viruses. Ten studies used the univalent vaccine containing the H1N1 virus-like strain. Only in one study were the SLE patients administered the bivalent H1N1/H3N2 vaccine. The vaccines were non-adjuvant split or subunit vaccines, except one that contained MF59 adjuvant. The interval between influenza vaccination and collection of blood samples was 3–6 weeks.

### Efficacy of influenza vaccine in SLE patients compared with healthy controls

The H1N1, H3N2 and B influenza vaccines were overall seroprotective in 68% 76% and 66% of patients with SLE, respectively; the crude rates of seroconversion were 57%, 53% and 42%, respectively. In almost all studies in which data were applicable, factors that increase antibody GMT >2.5-fold (Table 1). Only one study with the factors <2.5-fold showed a high level of mean antibody titer before influenza vaccination.

**Fig 2:**
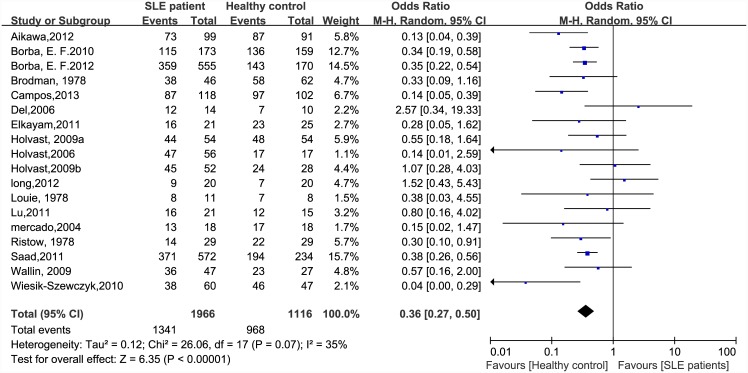
Forest plot of seroprotection rate of H1N1 vaccination in SLE patients compared with healthy controls.

**Fig 3:**
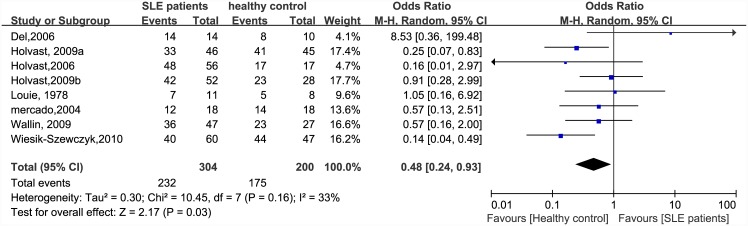
Forest plot of seroprotection rate of H3N2 vaccination in SLE patients compared with healthy controls.

**Fig 4:**
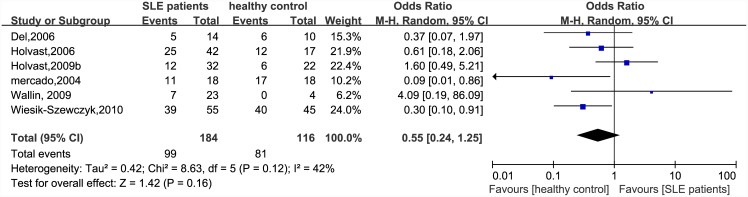
Forest plot of seroprotection rate of influenza B vaccination in SLE patients compared with healthy controls.

**Fig 5:**
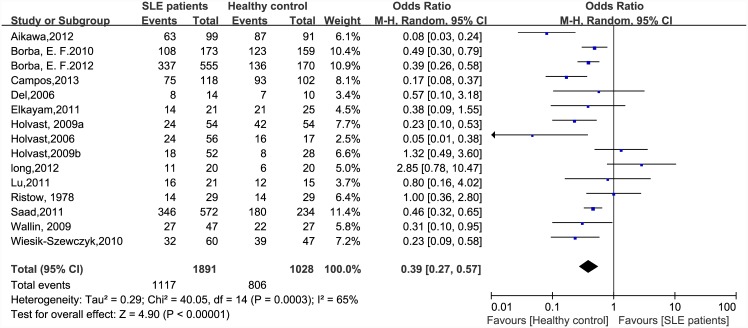
Forest plot of seroconversion rate of H1N1 vaccination in SLE patients compared with healthy controls.

**Fig 6:**
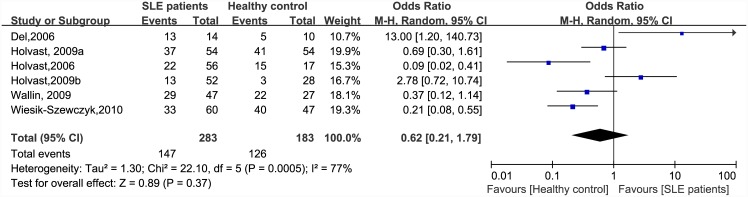
Forest plot of seroconversion rate of H3N2 vaccination in SLE patients compared with healthy controls.

**Fig 7:**
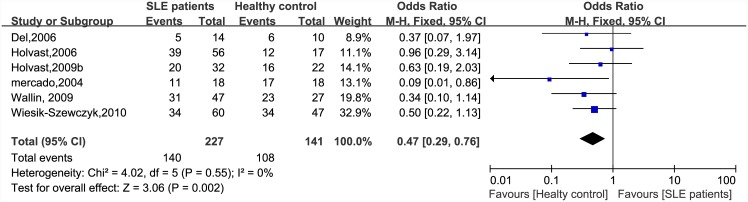
Forest plot of seroconversion rate of influenza B vaccination in SLE patients compared with healthy controls.

There were significant differences in immunogenicity between the patients and controls. The results are shown in the forest plot (Figs 2–7). With regards to the H1N1 vaccine, the OR for seroprotection was 0.36, (95%CI: 0.27–0.50), and the OR for seroconversion was 0.39 (95% CI: 0.27–0.57). For the H3N2 vaccine, there was only a significant difference in seroprotection rate (SLE patients versus healthy controls, OR = 0.48, 95% CI: 0.24–0.93), but not for seroconversion rate (OR = 0.62, 95% CI: 0.21–1.79). For the B-type influenza vaccine, the seroconversion rate (OR = 0.47, 95% CI: 0.29–0.76) also significantly decreased in patients with SLE. However, seroprotection for influenza B vaccination had a tendency to decrease (OR = 0.55, 95% CI: 0.24–1.25).

### Safety

**Fig 8:**
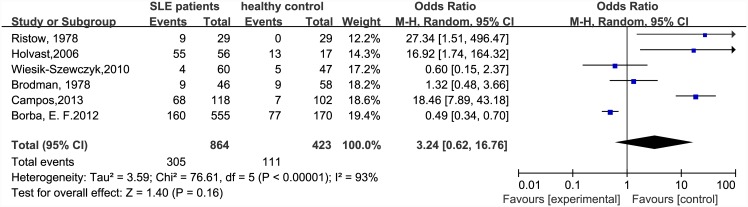
Forest plot of rate of side effects in SLE patients compared with the general population.

All side effects were mild and transient. The rate of side effects in the SLE patients was not significantly higher than that in the general population. The OR (SLE patients vs healthy controls) was 3.24 (95% CI: 0.62–16.76) (Fig 8). Among 1966 patients with SLE, 32 patients had mild SLE exacerbation and five had serious side effects. The latter included one death, two hospitalizations and two had severe SLE exacerbations.

### Publication bias assessment

All the evidence indicates that there was no publication bias in the meta-analysis.

## Discussion

Concerns about the safety and efficacy of vaccines in patients with SLE have existed over the past 30 years. However, the concerns have not been eliminated and this situation has impeded vaccine promotion. In this analysis, we found that the immunogenicity of influenza vaccination in SLE patients both compared and did not compare with that in healthy people. The pooled results showed that the efficacy of the influenza vaccine in SLE patients was significantly weaker than that in healthy controls, except for the seroconversion of H3N2 vaccine and the seroprotection of influenza B vaccine. The most likely interpretation for this exception is the limited number of studies in the meta-analysis. Although the efficacy of influenza vaccine in patients with SLE was weaker than that in the general population, the immunogenicity of the vaccine almost reached that in the CPMP guidelines. This means that influenza vaccine has a moderate effect on protecting patients with SLE. Considering that SLE patients are prone to suffer SLE exacerbation and serious complications after influenza infection, influenza vaccination could be a reasonable option to protect SLE patients during influenza pandemic periods.

The studies included in our meta-analysis showed that drugs such as glucocorticoids, azathioprine, methotrexate and mycophenolate mofetil potentially weakened humoral response to influenza vaccination. A review by McMahan and colleagues supported these findings. They reported that both biological and non-biological agents had a detrimental effect on vaccine immunity [32]. However, these results were not consistent with the data reported by Murdaca *et al*. [33]. One possible explanation for this disagreement is that they ignored SLE itself. The patients with SLE had a common feature of immunosuppressive therapy. The relationship between immunosuppressive therapy and SLE itself has been blurred. As a result, it is difficult to determine which factors really weaken the response to influenza vaccine. Further studies will be needed to detect the relationship between immunosuppressive therapy and SLE itself.

Age may play an important role in attenuating the efficacy of influenza vaccination. Long *et al*. found that the proportion of subjects (age <18 years) who responded to H1N1/2009 and trivalent influenza vaccine increased significantly with age [31]. The immunological response was also lower in elderly individuals [33]. We hypothesize that age may be related to vaccine efficacy. After age subgroup analysis, however, no significant difference was seen between patients age <18 and >18 years. In our included studies almost all patients were >6 years old. Healthy children (age >6 years) have normal immunological response to vaccine as compared to healthy adults. There were insufficient data in our study to understand the immunogenicity in elderly patients with SLE.

Another area of concern was the vaccine safety in SLE patients. In most of the studies, side effects were more frequent in patients than in the healthy controls. All side effects were mild and manageable. These symptoms were transient and not troublesome to vaccinated patients. Patients with SLE have a weakened sense of wellbeing. The higher frequency of mild side effects might have been a result of a reporting bias in patients [18].

Thirty-seven cases of disease exacerbation and serious adverse events were reported in 18 studies. In the study of Del Porto *et al*., the flares were not significantly different between vaccinated and non-vaccinated SLE populations [16]. Two of the 24 SLE patients with vaccination developed flares and one of the 24, who was not vaccinated, also showed flares. The symptoms of these patients were mild and moderate. One of the 21 SLE patients in the study of Lu *et al*. experienced a major change in disease activity and her SLEDAI score shift from 4 to 12 [22]. The patient’s vision became blurred after vaccination in the setting of being diagnosed with bilateral optic neuritis without neurological sequelae before vaccination. After pulse methylprednisolone therapy, neither blurred vision nor SLE flares developed in the following 6 months. Ristow and colleagues reported that two patients needed hospitalization for thrombophlebitis and a transient respiratory illness of probable infectious cause, and another patient with severe coronary atherosclerosis died from myocardial infarction 12 weeks after vaccination [23]. In a study with 62 SLE patients, exacerbation was seen in seven patients, and two of them underwent treatment adjustment. One was graded as severe because of the need for repeated treatment with cyclophosphamide. The other one was prescribed an increased dose of prednisone and additional chloroquine [26]. The seven flares, which occurred at 4–12 weeks after vaccination, comprised one severe flare for which SLEDAI shifted from 4 to 10 and six mild and moderate flares for which SLEDAIs fluctuated between 0 and 7. Eleven of 46 patients reported mild symptoms relating to SLE after influenza vaccination in the study of Brodman *et al*. [15]. Among 173 SLE patients in the study of Borba *et al*., SLEDAI increased in 12 SLE patients with a baseline score < 6 and in one patient whose SLEDAI was > 6 before vaccination [29]. It was not reported in other studies that the SLE disease activity changes and serious adverse events emerged during the term of observation.

Most of the serious adverse events could not be attributed to the influenza vaccine. There was lack of direct evidences to verify that influenza can trigger exacerbation and induce serious adverse events. Some patients with serious adverse events had other baseline diseases. Influenza vaccines produce immunological responses during the first few weeks following vaccination. If vaccination triggers disease exacerbation, it would be expected to happen particularly during this early period [18]. However, the duration between vaccination and occurrence of adverse events was >4 weeks in some reported cases.

The status of SLE disease at vaccination may influence the safety and efficacy of influenza vaccination. However, at lease 56% of the subjects were patients with low-to-moderate SLEDAI score or stable disease. There is not enough information to assess the safety and efficacy of influenza vaccination in SLE patients with unstable disease. Our data suggest that influenza vaccine is moderately effective and not detrimental to patients with a low-to-moderate SLEDAI score or stable disease.

## Conclusion

Although the humoral response to influenza vaccine in SLE patients was weaker than that in the general population, the immunogenicity in the former almost reached that in the CPMP guidelines. Influenza vaccine does not exacerbate SLE. Considering a higher risk of SLE exacerbation and a more severe course of infection among SLE patients, influenza vaccination is a feasible way to protect those patients during influenza pandemic periods. Further studies should be performed to explore how the immunosuppressive drugs and SLE itself influence the influenza vaccine response in SLE patients.

## Supporting Information

S1 File PRISMA 2009 Checklist. (DOC)
